# Response to: HCE-T cells express cornea-specific differentiation marker, PAX6 protein

**DOI:** 10.1007/s00417-022-05762-y

**Published:** 2022-07-25

**Authors:** Lorenz Latta, Tanja Stachon, Berthold Seitz, Nóra Szentmáry

**Affiliations:** 1 Dr. Rolf M. Schwiete Center for Limbal Stem Cell and Aniridia Research, Saarland University, Kirrberger Str. 100, 66424 Homburg, Saar Germany; 2 Department of Ophthalmology, Saarland University Medical Center, Homburg, Saar Germany

Dear Editor,

We thank Araki-Sasaki et al. for their important comments on our study.

The purpose of our experiments was to investigate to which extent the HCE-T cell line [1] is suitable for analysis of corneal epithelial cell differentiation. Therefore, we compared expression of different markers using qPCR and Western blot in the HCE-T cell line, in human primary limbal epithelial cells (LEC), and in differentiated primary human corneal epithelial cells (pCEC). Expression levels of conjunctival- and corneal-specific keratin and adhesion markers (KRT3, KRT12, KRT13, KRT19, DSG1), stem cell and differentiation markers (PAX6, ABCG2, ADH7, TP63, ALDH1A1), and additional (unvalidated) putative differentiation and stem cell markers (CTSV, SPINK7, DKK1) were examined with qPCR. Additionally, KRT3, KRT12, DSG1, and PAX6 protein levels were analyzed with Western blot [2].

The PAX6 measurement results showed a clear difference at mRNA and protein level between the HCE-T cell line, undifferentiated primary LEC, and pCEC. Although PAX6 mRNA and protein expressions were verifiable in the HCE-T cell line (especially using KSFM), these were detected at a much lower level than those in LEC and the pCEC [2]. This was an unexpected result for us.

The actual data presented by Araki-Sasaki is not quantitative. Therefore, it remains unclear whether our HCE-T cell batch has a lower PAX6 level compared to the original cell batch. In our hands, PAX6 expression could also be detected in the oral mucosa (unpublished data), by RT-PCR. Our statement that PAX6 protein is hardly detectable in the HCE-T cell line is a comparison to the expression levels in differentiated LEC and pCEC [2].

In our publication, we also aimed to point out the difference in expression level of several differentiation and stem cell markers between pCEC, LEC, and the HCE-T cell line [2]. Our study demonstrated a lower KRT3, KRT12, KRT13, KRT19 DSG1, ADH7, ALDH1A1, TP63, CTSV, and SPINK7 mRNA expression in the HCE-T cell line than that in differentiated LEC and in pCEC [2].

Due to the low PAX6 expression levels in the HCE-T cell line (compared to LEC and pCEC), we aimed to accentuate that the HCE-T cell line needs to be kept under special consideration, in case of its use for epithelial cell differentiation studies. In fact, the HCE-T cell line is a well-established model to analyze epithelial cell behavior [1, 3–6]. Neverthelesss, we aimed to point out carefully where limitations of a model like the HCE-T cell line might lie. The results of our study may of course differ slightly from results in other laboratories, as other types of antibodies may have been used.

It is extremely important for us to emphasize that we do not want to question that the HCE-T cell line is an appropriate model for studying human corneal epithelial cells in vitro, and we expressly apologize for any misunderstandings.
